# Characterization of a Compound Heterozygous SLC2A9 Mutation That Causes Hypouricemia

**DOI:** 10.3390/biomedicines9091172

**Published:** 2021-09-06

**Authors:** Jaeho Yoon, Raul Cachau, Victor A. David, Mary Thompson, Wooram Jung, Sun-Ha Jee, Ira O. Daar, Cheryl A. Winkler, Sung-Kweon Cho

**Affiliations:** 1 Cancer & Developmental Biology Laboratory, Center for Cancer Research, National Cancer Institute, Frederick, MD 21701, USA; jaeho.yoon@nih.gov (J.Y.); daari@mail.nih.gov (I.O.D.); 2 Advanced Biomedical Computational Science, Frederick National Laboratory for Cancer Research, National Cancer Institute, Frederick, MD 21701, USA; raul.cachau@nih.gov; 3 Molecular Genetic Epidemiology Section, Basic Research Laboratory, National Cancer Institute, Frederick, MD 21701, USA; davidvic@mail.nih.gov (V.A.D.); winklerc@mail.nih.gov (C.A.W.); 4 Center for Cancer Research, National Cancer Institute, Frederick, MD 21701, USA; thompsonm@mail.nih.gov; 5 Department of Cancer Biology, Lerner Research Institute, Cleveland Clinic, Cleveland, OH 44195, USA; JUNGW2@ccf.org; 6 Department of Epidemiology and Health Promotion, Institute for Health Promotion, Graduate School of Public Health, Yonsei University College of Medicine, Seoul 03722, Korea; shjee@yuhs.ac; 7 Department of Pharmacology, Ajou University School of Medicine, 164, Worldcup-ro, Yeongtong-gu, Suwon 16499, Korea

**Keywords:** *SLC2A9*, hypouricemia

## Abstract

Renal hypouricemia is a rare genetic disorder. Hypouricemia can present as renal stones or exercise-induced acute renal failure, but most cases are asymptomatic. Our previous study showed that two recessive variants of SLC22A12 (p.Trp258*, pArg90His) were identified in 90% of the hypouricemia patients from two independent cohorts: the Korean genome and epidemiology study (KoGES) and the Korean Cancer Prevention Study (KCPS-II). In this work, we investigate the genetic causes of hypouricemia in the rest of the 10% of unsolved cases. We found a novel non-synonymous mutation of *SLC2A9* (voltage-sensitive uric acid transporter) in the whole-exome sequencing (WES) results. Molecular dynamics prediction suggests that the novel mutation p.Met126Val in *SLCA9b* (p.Met155Val in *SLC2A9a)* hinders uric acid transport through a defect of the outward open geometry. Molecular analysis using *Xenopus* oocytes confirmed that the p.Met126Val mutation significantly reduced uric acid transport but does not affect the *SLC2A9* protein expression level. Our results will shed light on a better understanding of *SLC2A9*-mediated uric acid transport and the development of a uric acid-lowering agent.

## 1. Introduction

The homeostasis of serum uric acid (SUA) levels can be achieved by the dynamic processes of production and elimination. Hypouricemia, with a low uric acid level, is defined as an SUA concentration < 2 mg/dL. Hypouricemia is generally asymptomatic in the general population; most hypouricemia patients are identified by chance in regular health examinations [1]. Renal hypouricemia (RUHC) is a rare genetic disorder diagnosed by hypouricemia and increased fractional excretion of uric acid (UA > 10%) [2]. There are two types of RUHC: *SLC22A12* mutations cause type 1 RHUC (OMIM: 220150), and *SLC2A9* mutations cause type 2 RHUC (OMIM: 612076). Unlike hypouricemia, RHUC is sometimes accompanied by severe complications, such as exercise-induced acute kidney injury (EIAKI) and urolithiasis [3]. In the case of urolithiasis, RUHC is 6–7 times more prevalent than in those with normal levels of SUA [4].

The prevalence of hypouricemia is 0.41% in Koreans [5] which is similar to the prevalence in the Japanese (0.46%) [6]. This may be caused by a founder mutation, the protein-truncating p.Trp258* mutation of *SLC22A12* [7]. Population-specific mutations have also been identified in different ethnic groups. Rare missense variants (p.R325W, p.R405C, and p.T467M) of *SLC22A12* were reported in European and African American populations [8]. Various ethnic groups, including Israeli-Arab, Iraqi-Jewish, and Roma populations also harbor deleterious mutations of *SLC22A12* and *SLC2A9* [2,9,10,11,12,13].

In our previous study, approximately 90% of hypouricemia patients showed two variants in *SLC22A12,* p.W258* and p.Arg90His, in two independent cohorts, the Korean Genome and Epidemiology Study (KoGES, *n* = 179,318) and the Korean Cancer Prevention Study (KCPS-II, *n* = 156,701) [14]. However, the seven hypouricemia cases did not present the Asian-specific variants *SLC22A12* p.W258* (rs121907892) and p.Arg90His (rs121907896), suggesting that other genes are also involved in the regulation of serum uric acid (SUA) levels.

In this study, we identify a novel variant in *SLC2A9* by whole-exome sequencing (WES) of the unsolved cases of hypouricemia. *SLC2A9* was initially identified as a glucose transporter that regulates the homeostasis of glucose levels. However, recent studies have demonstrated that *SLC2A9* transports uric acid, and that genetic mutations in *SLC2A9* have been linked to hyperuricemia and gout [15,16]. Our in silico and in vitro assay suggest that the novel mutation p.Met126Val in *SLCA9b* significantly reduces urate transport, resulting in hypouricemia.

## 2. Methods

### 2.1. Study Participants

This study was approved by the institutional review board of Sungkyunkwan University (IRB# SKKU 2017-12-007) and the Korean Cancer Prevention Study (KCPS-II) cohort from the Severance Hospital, Seoul, Korea (IRB#4-2011-0277) (approval date: Date, February, 2011) [17]. Whole-exome sequencing (WES) was performed in 7 patients with unexplained hypouricemia.

### 2.2. DNA Preparation and Whole-Exome Sequencing

Genomic DNA was obtained from peripheral blood leukocytes. DNA quality and quantity were assessed by an OD260/280 ratio of 1.8–2.0, 1% agarose gel electrophoresis, and PicoGreen^®^ dsDNA Assay (Invitrogen, Waltham, MA, USA). SureSelect sequencing libraries were prepared (Agilent SureSelect All Exon kit 50 Mb, Santa Clara, CA, USA) and the enriched library was sequenced using the HiSeq 2500 sequencing system (Illumina, San Diego, CA, USA). Image analysis and base calling were performed with the pipeline software using default parameters. Mapping was completed using the human reference genome assembly (GRCh37/hg19), and all variants were called and annotated using the CLC Genomic Workbench (version 9.0.1) software (QIAGEN bioinformatics, Redwood City, CA, USA).

### 2.3. WES Variant Filtering Analysis

The overall variant-identifying process referred to the standard guidelines of investigating variants for Mendelian disorders from WES data [18,19]. We performed the analysis, assuming an autosomal recessive or X-linked recessive pattern according to the observed inheritance mode in hereditary RHUC [20]. First, based on the prevalence of hypouricemia without other medical conditions, such as hypertension or diabetes mellitus (31/179,318), the Hardy-Weinberg equation was used to calculate the allele frequency threshold to 0.01 and we excluded variants with MAF > 1% in the dbSNP database (version 150), 1000 Genome Projects phase 3 data (2504 individuals), and Genome Aggregation Database (gnomAD, http://gnomad.broadinstitute.org/ accessed date: 27 August 2020) [21]. Second, variants present in the homozygous or hemizygous state in 46 healthy Koreans without hypouricemia were excluded. Third, non-synonymous variants, insertion/deletion (indel), or splice-site variants were selected. In the further analysis, we excluded single heterozygous variants so that homozygous variants and putative compound heterozygous variants finally remained. For males, hemizygous variants in the X chromosome were considered to be retained. The numbers of variants are listed in Table S3.

### 2.4. Direct Sanger Sequencing

Confirmation of called variants was conducted via direct Sanger sequencing. The DNA sequences spanning the variants were amplified using specific primers (Table S4) and sequenced using an Applied Biosystems genetic analyzer 3500XL (Applied Biosystems, Foster City, CA, USA).

### 2.5. In Silico Analysis of Novel Missense Variants and Molecular Dynamics

#### 2.5.1. In Silico Prediction

Prior to the analysis, known pathogenic variants of *SLC2A9* were screened in the Human Gene Mutation Database (HGMD). For the newly discovered missense *SLC2A9* and other candidate variants, we queried whether mutated amino acid residues were highly conserved across the vertebrate orthologs, using the UCSC Genome Browser (https://genome.ucsc.edu/; accessed date: 27 August 2020). Given the functional role of nitrogen excretion in the evolutionary process, we identified amino acid sequences in several mammals (*Rhesus macaque, Mus musculus* and *Canis lupus familiaris*) Third, the prediction of the functional effect of missense variants was performed using the latest version of PolyPhen-2 (http://genetics.bwh.harvard.edu/pph2; accessed date: 27 August 2020), SIFT (sorting intolerant from tolerant, http://sift.jcvi.org; accessed date: 27 August 2020), Condel (consensus deleteriousness score of non-synonymous single nucleotide variants, http://bbglab.irbbarcelona.org/fannsdb; accessed date: 27 August 2020), and the mutation taster (http://www.mutationtaster.org; accessed date: 27 August 2020) algorithms [22,23,24,25].

#### 2.5.2. Molecular Dynamics

**Figure 1:**
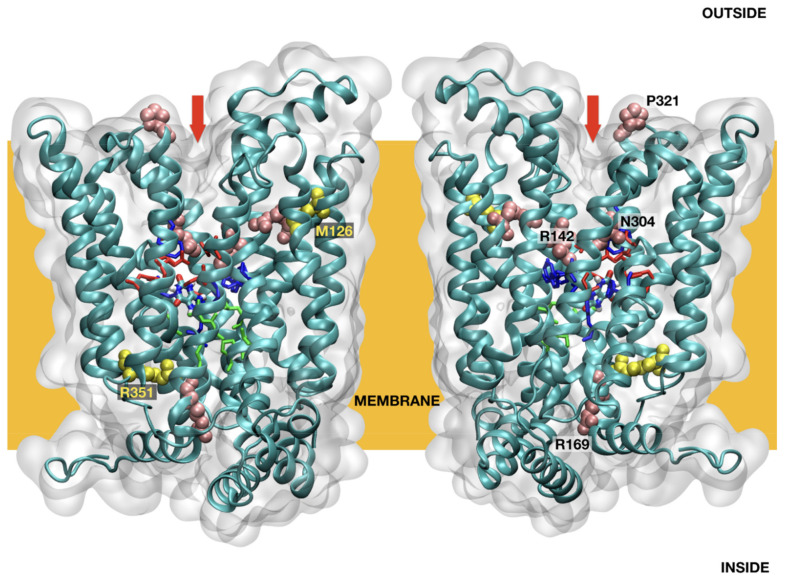
*SLC2A9b* model structure overview.

All homology models of *SLC2A9* were combined using feedback restrained molecular dynamics [26,27] to form a consensus model (Figure 1). FRMD affords a simple protocol to maximally retain structural features during a molecular dynamics trajectory while minimizing the distortions imposed by an external restraint. All molecular dynamics calculations were performed with NAMD, using an Amberff99SB force field in the NVT ensemble at typical settings (T = 298 K, 2fs integration time, 12A cutoffs), as obtained using QwikMD in VMD with default parameters to prepare the input files [28]. The molecular dynamics results reported are from 125 ns trajectories unless otherwise stated. The overall organization of *SLC2A9* is similar to that described by Clemencon [29] (Figure 1). The mutation sites do not cluster in any obvious arrangement or loci, nor do the mutated residues appear to follow a simple distribution pattern (Figure 1). A qualitative evaluation of the mutation effect was made, based on some simple criteria. Mutations affecting the binding site or the entry/exit channel section of the model suggest a direct effect on transport. Mutations resulting in structural changes may affect transport indirectly, by either changing the shape of the transporter or impending its dynamic rearrangement as required for transport (Table 1). The structural effect was evaluated as an increase in the root mean square displacement (RMSD) deviation, computed during 25 ns of molecular dynamics (after 25 ns of equilibration), measured against the conformations obtained during a 25 ns trajectory for the initial sequence.

#### 2.5.3. Molecular/Functional Studies

##### Generation of SLC2A9b Expression Vectors

The recombinant plasmid *SLC2A9b*-eGFP/pcDNA-DEST47 [30] was a gift from Wolf Frommer (Addgene plasmid # 18730; http://n2t.net/addgene:18730, accessed date: 27 August 2020; RRID:Addgene_18730). To generate the SLC2A9b-V5 expression vector, the SLC2A9b-V5 DNA fragment was inserted into the pCS107 vector after PCR amplification with appropriate primers as follows, with *SLC2A9b* forward: 5′-GGCCATCGATAGCC ACCATGAAGCTCAGTAAAAAGGACCGAGGAGAAGATGAAGAAAGTGATTCAGCG-3′; and *SLC2A9*-V5 reverse: 5′-GCCTGCGGCCGCTTACGTAGAATCGAGACCGAGGAGAGGGTTAGGGATAGGC TTACCAG GCCTTCCATTTATCTTACCATCAG-3′. The amplified DNAs were assembled into the pCS107 vector using T4 DNA Ligase (M0202S, NEB) after ClaI and NotI endonuclease treatment. Then, 10 µL of the ligase reaction mixture was transformed into 120 µL of chemically competent DH5α (18258012; Thermo Fisher Scientific, Waltham, MA, USA) cells and screened on ampicillin-containing LB plates.

##### Site-Directed Mutagenesis for the Met126Val Mutant

To generate a site-specific point-mutation of *SLC2A9b*-Met126Val, the QuikChange II Site-Directed Mutagenesis Kit (200524; Agilent Technologies, Santa Clara, CA, USA) was used with appropriate primers, as follows, with Met126Val forward: 5′-GAGCGAGCAGGCCACCAGCAATGCAGCAG-3′; Met126Val reverse: 5′-CTGCTGCATTGCTGGTGGCCTGCTCGCTC-3′. Confirmation of the introduction of the Met126Val mutation into the vector was confirmed by Sanger sequencing. Plasmid DNA was purified for transfection and oocyte experiments using a GenElute endotoxin-free plasmid maxiprep kit (NA0310; Sigma-Aldrich, Burlington, MA, USA).

##### In Vitro Transcription

All mutants and wild-type cDNAs were linearized by Asp718 (Roche). In vitro transcription was performed with SP6 mMESSAGE mMACHINE Kit (AM1340, Thermo Fisher Scientific, Waltham, MA, USA).

##### *SLC2A9b* Expression in *X. laevis* Oocytes

Stages V–VI oocytes were collected from *X. laevis* (Nasco, Chicago, IL, USA). Oocytes were injected with 10 ng of either wild-type or mutant RNAs or the equivalent volume of water, and incubated for 48 h in oocyte medium (50% L-15 + glutamine, 40% HEPES/insulin stock, 10% fetal calf serum, 100 mg/mL gentamycin). Animal care and use for this study were performed in accordance with the recommendations of AAALAC for the care and use of laboratory animals in an AAALAC-approved facility. Experimental procedures were specifically approved by the animal care and use committee of the National Cancer Institute-Frederick ASP #18-433, in compliance with AAALAC guidelines.

##### Western Blot Analysis

Using ten oocytes of the experimental groups, lysates were prepared with ice-cold TNSG buffer (20 mM Tris-HCl pH 7.5, 137 mM NaCl and 1% NP-40). The lysates were separated on a 10% SDS polyacrylamide gel and transferred onto a PVDF membrane (88025, Thermo Fisher Scientific, Waltham, MA, USA). The membranes were incubated overnight with anti-mouse V5-HRP monoclonal antibody (R961-25, Thermo Fisher Scientific, Waltham, MA, USA) (1:2000) after blocking with 10% skim milk. The SLC2A9b signal was revealed by ECL (32106, Thermo Fisher Scientific, Waltham, MA, USA) and exposed on Kodak films.

##### Uric Acid Uptake Assay

10 ng mRNA of SLC2A9-wild type or mutants were injected into the cytoplasm, at the midline of stage VI *Xenopus laevis* oocytes defolliculated with collagenase (2.5 mg/mL). Two days after injection, we performed the uric acid uptake assay at room temperature in ND-96 buffer (96 mM NaCl, 2 mM KCl, 1.8 mM CaCl2, 1 mM MgCl2, 5 mM Hepes, pH 7.4) for 120 min. We used 100 mM [^14^C] of uric acid (ARC 0513A-50 µCi, American Radiolabeled Chemicals, St. Louis MO, USA) to assess urate uptake. Following five washes in ND-96 lacking radiolabel, ten oocytes of experimental groups were collected, lysed in 200 mL of 10% SDS, and subjected to scintillation counting. We harvested ten pools of ten oocytes per experimental point. Statistical data analyses were performed using Prism 8.

##### Confocal Microscopy

Briefly, the oocytes were fixed in 4% paraformaldehyde in PBS overnight at 4 °C. The oocytes were then embedded in 4% low-melting agarose gel and were sectioned to a thickness of 100 μm with the vibratome (LEICA VT 1200S). The primary antibodies, anti-V5 (1:500, G189, ABM) and the secondary antibodies, anti-mouse Alexa Fluor 594 (A32744, Invitrogen) were incubated at 4 °C overnight. The samples were washed, mounted and imaged using a Zeiss LSM-880 laser-scanning confocal microscope.

## 3. Results

### 3.1. Demographics

Baseline patient characteristics are summarized in Table 1. The 7 sequenced participants had unsolved hypouricemia (UA 0.79 ± 0.47 mg/dL; age 42 ± 11 years; BMI 24.9 ± 2.7 kg/m^2^; total cholesterol level 209 ± 35 mg/dL) that were not due to known causes (e.g., SUA-lowering drugs).

#### 3.1.1. Identification of Novel Variants in *SLC2A9b* by Whole-Exome Sequencing

WES analysis was performed on 7 subjects identified from the KoGES and KCPS-II cohorts as previously described [14], with an average depth of coverage of 85-fold. We performed variant calling and downstream filtering analyses, assuming an autosomal recessive inheritance or sex-linked hemizygous patterns. One individual (NIH17A8568242) carried compound heterozygous variants (p.Met155Val (c.463A > G, exon 5), p.Arg380Gly (c.1138C > T, exon 10)) of *SLC2A9a* (long form). The corresponding residues for *SLC2A9b* (short form) are p.Met126Val and p.Arg351Gly, respectively. p.Arg380Gly was previously reported by HGMD (The Human Gene Mutation Database). The p.Met155Val variant was confirmed by Sanger sequencing (Supplementary Figure S1). The global minor allele frequency of the novel variant is 0.000022 at the gnomAD database (http://gnomad.broadinstitute.org; accessed date: 27 August 2020).

In the remaining six individuals, we found 12 candidate genes for unexplained cases (8 genes for homozygous: *ATP8B2, KRTAP5-8, PIK3CB, ASIC3, ADAM8, RBM12, PWWP2B, SULT1A2*; 4 genes for hemizygous: *ASB12, RLIM, GPR101, PPEF1*) The possible disease-causing variants are listed in Supplementary Table S1 (recessive mode). In a systematic review, most of the genes were not found to be involved in biological pathways affecting UA levels. However, the p.Arg78His variant (rs145118752) of *ASB12* on chromosome X, which was discovered in two cases, NIH17A8004492 and YID182829, was found in 0.018% of the global population and 0.16% when limited to East Asia only (gnomAD 2020.11, http://gnomad.broadinstitute.org/; accessed date: 27 August 2020). We also found that these two individuals are unrelated (kinship coefficient = 0.001).

#### 3.1.2. In Silico and Molecular Dynamics Prediction of *SLC2A9b*

The functional prediction for the novel variant of *SLC2A9b*, p.Met126Val, is predicted as pathogenic by using mutation taster, PolyPhen-2, SIFT and CADD (disease-causing, damaging, deleterious, and 18.24, respectively). The amino acid is highly conserved across vertebrate species down to zebrafish.

#### 3.1.3. Molecular Dynamics Prediction of *SLC2A9b* and Its Affinity for Uric Acid

**Figure 2:**
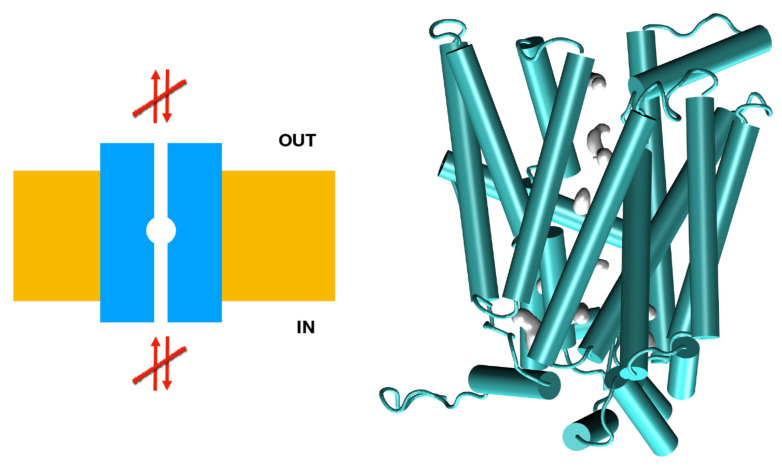
Mechanistic interpretation of the effects of the M126V mutation of *SLC2A9b*. The M126V model suggests that this mutation renders the vestibular regions unavailable. Left: schematic representation of the channel (in blue), membrane (yellow), and flow (arrows). Right: a snapshot of the M126V model during an MD trajectory, in cartoon representation in green. Internal space is represented by showing the solvent’s accessible surface in gray.

The consequence of the amino acid substitution in *SLC2A9b* was investigated using a molecular dynamic prediction analysis (Figure 1). Molecular dynamics simulations of the p.Met126Val mutant model suggest the mutation of Met126 to Val126 results in compacting of the helical bundle, resulting in an extremely stable arrangement, with RMSF values for the region surrounding the Val126 residue being 30% lower than those observed in the reference model (Figure 2). This stable arrangement may explain the unexpectedly large effect of this seemingly inconsequential mutation in the transporter’s overall function, stiffening the vestibular areas occluding the binding pocket, and resulting in a less functional rocker structure.

**Figure 3:**
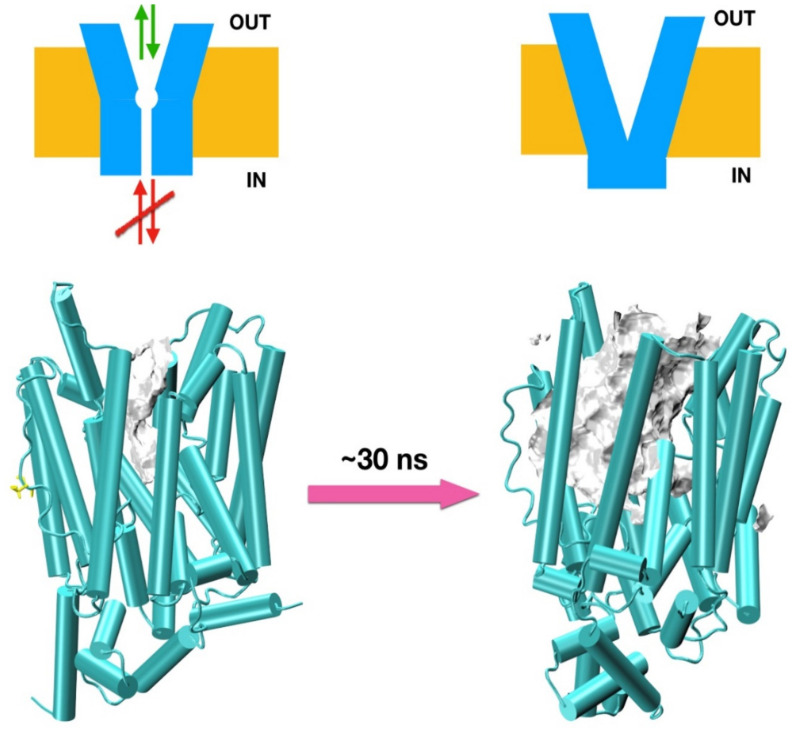
Effects of the R351W mutation of *SLC2A9b*. Top: schematic representation of the channel (in blue), membrane (yellow), and flow (arrows). Bottom: snapshots of the R351W model during an MD trajectory in cartoon representation in green. Internal space is represented by showing the solvent accessible surface in gray (compared to similar surfaces describing the vestibular areas in Figure 2). The initial model structure is surprisingly stable, but it deforms under molecular dynamics simulations, as seen in the snapshot on the right after ~30 ns of MD trajectory. Notice the very substantial reorganization of the internal helices.

Molecular dynamics simulations of the p.Arg351Trp model suggest the mutation of Arg351 to Trp351 breaks a well-structured chain of 12 charged resides including Lys, Arg, Tyr, and Glu spanning over 20 A (Figure 3) and stabilizing the intracellular domain. This polar structure plays a crucial role in directing anions to the intracellular vestibular area. The p.Arg351Trp mutation affects the inside binding site, decreasing the ∆B1 binding energy of urate to −1.2 Kcal/mol, possibly affecting urate transport. The dislodging of this domain may have an impact on the binding of external effectors as well. Energy barriers for 2 variants are illustrated in Table S2.

### 3.2. Molecular Analysis

#### 3.2.1. SLC2A9b-p.Met126Val Expression Analysis in *X. laevis* Oocytes

**Figure 4:**
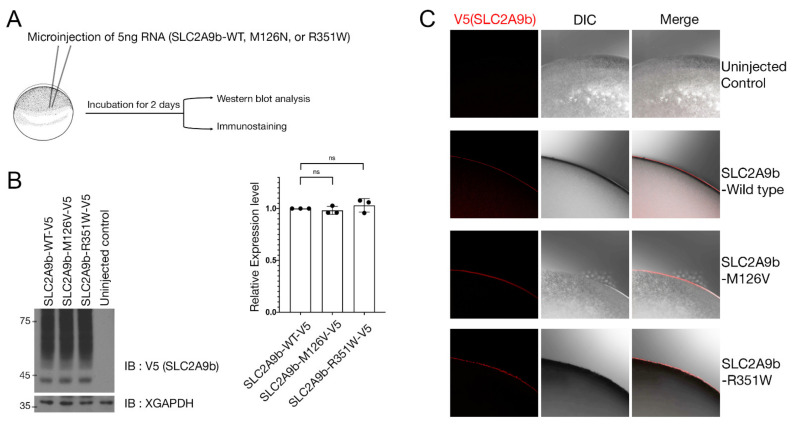
Expression of WT and mutant *SLC2A9b* in *Xenopus* oocytes. (**A**) Schematic representation of the experimental procedure. The same amount of wild-type or mutants *SLC2A9b* RNAs were injected into oocytes. The oocytes were harvested after 2 days and then, Western blot analysis or immunostaining was performed. (**B**) *SLC2A9b* wild type and mutants showed similar protein expression levels. The histogram depicts the relative protein expression level (*n* = 3). Quantification with one-way ANOVA (Dunnett’s multiple comparisons test), *p* = 0.4363. Data represent the mean ± S.D. of three individual experiments. *ns*: no statistical differences between the groups. (**C**) Immunostaining was performed using anti-V5 antibodies. Both WT and mutants *SLC2A9b* showed plasma membrane localization in *Xenopus* oocytes. DIC; differential interference contrast image.

*SLC2A9* has two isoforms, a long isoform (*SLC2A9a*) and a short isoform (*SLC2A9b*), that differ in their N-terminal region and plasma membrane localization. *SLC2A9a* localizes to the basolateral side of the plasma membrane, while *SLC2A9b* traffics to the apical side [31,32]. Previous studies have demonstrated that the basolateral *SLC2A9a* is involved in uric acid efflux, while the apical *SLC2A9b* plays a role in uric acid absorption [33,34,35]. To investigate whether the novel exonic mutation, p.Met126Val, affects the molecular function of *SLC2A9b*, the oocyte expression system was utilized. *Xenopus* oocytes are known as an excellent tool to study the molecular function of *SLC2A9* [29]. We generated the V5 tagged as the *SLC2A9b* p.Met126Val variant, corresponding to the p.Met155Val of *SLC2A9a*. One of the well-known exonic variants, p.Arg351Trp, corresponding to p. Arg380Trp of the *SLC2A9a*, was employed as a positive control [36]. To examine whether the p.Met126Val exonic mutation causes any change in the expression level of *SLC2A9b*. mRNAs of the *SLC2A9b* wild-type, M126V, and R351W were generated by in vitro transcription, and then, 5ng of each mRNA were injected into *Xenopus* oocytes. After 2 days of incubation to allow the translation of injected mRNA, Western blot analysis and immunostaining were performed (Figure 4A). Western blot analysis revealed no difference in the protein expression level of the exonic variants compared to the wild-type *SLC2A9b* (Figure 4B). Since *SLC2A9* is a membrane protein, and the plasma membrane localization influences the *SLC2A9* function, we investigated the subcellular localization of the *SLC2A9b* p.Met126Val variant using immuno-staining. Confocal microscopy analysis revealed that *SLC2A9b*-WT, *SLC2A9b*-p.Met126Val-V5 and *SLC2A9b*-p.Arg351Trp-V5 mutants were localized at the plasma membrane in *Xenopus* oocytes (Figure 4C).

Our results suggest that the novel exonic mutation, p.Met126Val, shows similar protein expression level subcellular localization compared to the wild type.

#### 3.2.2. Urate Transport Activity of SLC2A9b-p.Met126Val in Xenopus Oocytes

**Figure 5:**
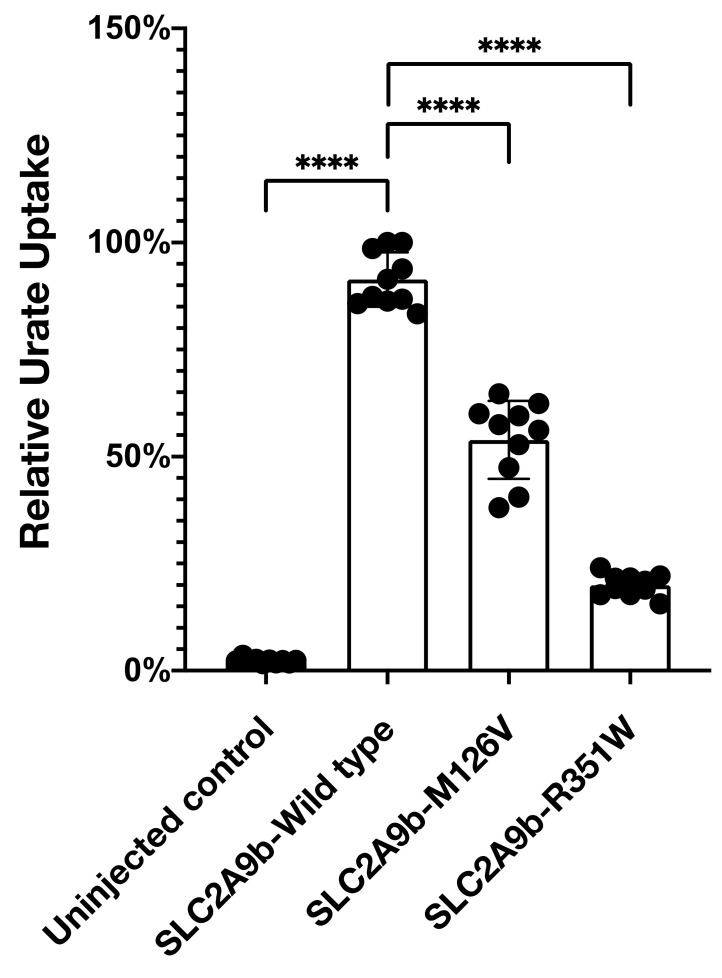
Met126Val mutation reduces uric acid uptake. Uric acid uptake assay was performed as described in the Methods section. M126V mutation in *SLC2A9b* reduced uric acid uptake by 45% and R351W mutation decreased by 70%. Histogram depicts relative urate uptake level (*n* = 10). Quantification with one-way ANOVA (Dunnett’s multiple comparisons test), **** *p* < 0.0001. Data represent the mean ± S.D. of three individual experiments. **** *p* < 0.0001.

Next, we analyzed urate transport activity using [^14^C]-uric acid as a substrate in *Xenopus* oocytes. Our result showed that the well-known exonic mutation, p.Arg351Trp, decreased uric acid transport activity by 70% compared to the wild type. Interestingly, the p.Met126Val mutation also reduced uric acid transport activity by 45% at 100 mM [^14^C]-uric acid concentration (Figure 5).

Our result suggests that our novel exonic mutation, p.Met126Val, may also contribute to hypouricemia.

## 4. Discussion

In this study, we comprehensively evaluated the contribution of *SLC2A9* to severe hypouricemia by first identifying variant (p.Met126Val) using WES, followed by molecular dynamics prediction and functional validation.

With regard to *SLC2A9b*, the p.Met126Val variant was identified in the case of NIH17A8568242 as a compound heterozygote with p.Arg351Trp. Molecular dynamics analysis supported a loss-of-function role considering its RMSD value reflecting structural changes in protein flexibility. Two missense mutations (p.Arg351Trp, rs121908321, and p.Arg169Cys, rs121908322 of *SLC2A9*b) are well-documented as causal for type 2 RHUC. Our experiments with *Xenopus* oocytes showed that p.Met155Val for the *SLC2A9a* (p. Met126Val for *SLC2A9b)* variant in *SLC2A9* causes a defect in uric acid transport. This is consistent with the individual who presented SUA levels that were near 0. *SLC2A9* is the most frequently reported gene associated with SUA levels, along with *ABCG2,* in GWAS studies of hyperuricemia and gout [37]. Intronic SNPs (rs4529048, rs7674711, and rs11936395) of *SLC2A9* have been associated with both increased SUA levels and increased risk of gout [38,39]. However, the missense variant (p.Val253Ile, rs16890979) of *SLC2A9* has been reported both as a protective SNP for gout and in lower UA levels [40,41]. Moreover, *SLC2A9* showed a statistically significant gene–gene interaction, with variants in the intergenic region located 80 kb downstream (*WDR1*-*ZNF518B*) [42]. A comprehensive study is needed to evaluate the effect of different transcriptional factors and the variation in regulatory elements on the gene expression of *SLC2A9*. Recently, large-scale WES using 19,517 participants (15,821 of European ancestry and 3696 of African ancestry) identified variants of *SLC22A12 and SLC2A9* that were associated with lower levels of SUA. Identified polymorphisms in uric acid transporter genes associated with lowering UA differ by ethnic group, due to a combination of founder effects, population isolation, and random drift. Collaborative international research with established cohorts, with GWAS and SUA measures, using a multi-ethnic approach is needed to explain the missing heritability of SUA and to further our understanding of the genetic architecture of SUA levels.

The two isoforms of *SLC2A9* differ at their N-terminal regions due to binding by the transcriptional factors to different promoters [35]. Both isoforms increase uric acid uptake when overexpressed in HEK293 cells and *Xenopus laevis* oocytes, with a peak UA uptake as early as 20 min upon uric acid incubation [43]. The overexpression of the *SLC2A9* mutant isoforms *SLC2A9a*-Leu75Arg (*SLC2A9b-*Leu46Arg) in oocytes resulted in a reduced uric acid uptake when compared to the reference protein [43]. However, *SLC2A9b-*Leu46Arg showed a greater reduction (80% decrease) in UA uptake than that of *SLC2A9a*-Leu75Arg (60%) [35]. Our in vitro studies show that the overexpression of the *SLC2A9b*-reference is sufficient to raise the intracellular concentration of UA when treated for 2 h. Throughout both of our in silico and in vitro studies, we showed that p.Met126Val of *SLC2A9b* is a causing variant for hypouricemia. One limitation of our approach is that we could not evaluate the change of uric acid binding affinity once it was transported into the cell. What we measured was the final concentration of uric acid at the equilibrium point. The change of binding affinity inside the membrane can be answered in future studies using a patch-clamp and electrophysiological evaluation.

We also identified two males with extreme hypouricemia who carried the X-linked *ASB12* variant of unknown significance. Little is known about the functional significance of this gene for uric acid transport. Since it is postulated that the ASB family may be involved in protein degradation via mediating the ubiquitin-proteasome system or signal transduction [44], it may be involved in the trafficking or intracellular degradation of the UA transporter. One limitation of this study is that family members were not available for the analysis of genotype-phenotype segregation through multiple generations. Given that known causative genes for hypouricemia remain unidentified, the genetic inheritance of hypouricemia could be more common than was indicated by our results.

## 5. Conclusions

We described the clinical and molecular characteristics of hypouricemia, caused by compound heterozygous mutations of *SLC2A9*. Our clinical and molecular findings may contribute to the understanding of the physiology of renal uric acid. We also proposed candidate genes for hypouricemia from unexplained cases for further study.

## Figures and Tables

**Table 1:** Demographic characteristics.

Characteristics	Unexplained Group	*SLC2A9* Compound Heterozygote
	*n* = 6	*n* = 1
Age (years)	43 ± 12	40
BMI ^†^ (kg/m^2^)	25.1 ± 2.9	23.8
Waist circumference, cm	81 ± 5	72
Blood pressure, mmHg		
Systolic	110 ± 3	124
Diastolic	71 ± 12	64
Smoking status		
Never a smoker, no. (%)	1 (16.67)	1 (100)
Ever a smoker, no. (%)	5 (83.33)	0 (0)
Alcohol consumption		
Never a drinker, no. (%)	2 (33.33)	1 (100)
Ever a drinker, no. (%)	4 (66.67)	0 (0)
Uric acid, mg/dL	0.78 ± 0.52	0.80
Total cholesterol, mg/dL	214 ± 34	174
Triglycerides, mg/dL	169 ± 69	178
Fasting glucose, mg/dL	86 ± 14	92
LDL cholesterol, mg/dL	116 ± 22	90.4
HDL cholesterol, mg/dL	64 ± 18	48
Creatinine, mg/dL	0.80 ± 0.25	0.70

Values are mean ± standard deviation (SD) for continuous data. ^†^ The body mass index (BMI) was calculated as weight in kilograms divided by height in meters squared.

## Data Availability

Data presented in this study are available on request from the corresponding author.

## Supplementary Materials

The following are available online at https://www.mdpi.com/article/10.3390/biomedicines9091172/s1, Supplementary Table S1: Possible variants identified in 6 individuals with hypouricemia by WES; Supplementary Table S2: Predicted functional impacts of amino acid changes of SLC2A9b; Supplementary Table S3: Variant filtering process or unsolved cases; Supplementary Table S4: Primer information for rs369512758 (SLC2A9); Supplementary Figure S1: Sanger confirmation of the novel variant p.M126V of SLC2A9b.
