# Supplementary material for: Characterization of a Compound Heterozygous SLC2A9 Mutation That Causes Hypouricemia
Source: Biomedicines. 2021 Sep 6;9(9):1172. doi: 10.3390/biomedicines9091172 (PMC8471325; doi:10.3390/biomedicines9091172)

Supplementary Table S1. Possible variants identified in 6 individuals with hypouricemia by WES.

Abbreviations are as follows: Chr, chromosome; Bn, benign; Condel, consensus deleteriousness score of non-synonymous single nucleotide variants; Dam, damaging; DC, disease causing; Del, deleterious; Neu, neutral; PM, polymorphism; PP2, PolyPhen-2 prediction score Humvar; SIFT, sorting Intolerant from Tolerant; SNP, single nucleotide polymorphism; Tol, tolerant.

[a]cDNA mutations are numbered according to human cDNA reference sequence NM_144585.2 (*URAT1*), NM_001001290.1 (*GLUT9*). [b]dbSNP database (http://www.ncbi.nlm.nih.gov/SNP). [c]gnomAD browser (http://gnomad.broadinstitute.org/). [e]Mutation taster (http://www.mutationtaster.org/). [f]PolyPhen-2 prediction score HumVar ranges from 0 to 1.0; 0 = benign, 1.0 = probably damaging (http://genetics.bwh.harvard.edu/pph2/). [g]SIFT (http://sift.jcvi.org/). [h]Condel (http://bbglab.irbbarcelona.org/fannsdb/). [i]CADD (http://cadd.gs.washington.edu/).

Individual	Gene Symbol	Muta-tion Type	Chr	Base position	Nucleotide change[a]	Amino acid change	Amino acid Conservation				Frequency in the dbSNP database[b]	Frequency in the gnomAD database[c]	Mutation Taster[e]	PP2 Humvar[f]	SIFT[g]	Condel[h]
							Rhesus	Mouse	Dog	Elep-hant						
NIH17A8004492	*HMCN1*	Comp Het	1	186043907	c.8174C>T	p.Ala2725Val	Ala	Ala	Ala	Ala	rs139140123<br>0.00005/5 (ExAC)<br>0.00008/1 (GO-ESP)	0.000044<br>(no hom)	DC	Dam<br>(0.636)	Del<br>(0.02)	Del<br>(0.606)
			1	186057881	c.9721A>G	p.Ile3241Val	Ile	Ile	Ile	Ile	rs138928800<br>0.0010/120 (ExAC)<br>0.0016/8 (1000G)<br>0.0002/2 (GO=ESP)<br>0.0001/3 (TOPMED)	0.001162<br>(no hom)	DC	Bn<br>(0.326)	Tol<br>(1)	Neu<br>(0.102)
	FAT4	Comp Het	4	126238958	c.1392T>A	p.Phe464Leu	Phe	Phe	Phe	Phe	rs767391145<br>0.000008/1 (ExAC)	0.000008<br>(no hom)	DC	Dam<br>(0.989)	Tol<br>(0.49)	Neu<br>(0.450)
			4	126371027	c.8856A>G	p.Ile2952Met	Ile	Ile	Ile	Ile	rs770138735<br>0.000008/1 (ExAC)	0.000016<br>(no hom)	DC	Dam<br>(0.999)	Del<br>(0.02)	Del<br>(0.873)
	ZNF143	Comp Het	11	9495485	c.206-3T>C	Splice site mutation					rs749661663<br>0.00002/2 (ExAC)	0.000008<br>(no hom)	-	-	-	-
			11	9546800	c.1700C>G	p.Ala567Gly	Ala	Ala	Ala	Ala	rs77240493<br>0.0001/15 (ExAC)<br>0.0006/3 (1000G)	0.000097<br>(no hom)	DC	Dam<br>(0.935)	Del<br>(0)	Del<br>(0.825)
	PPP6R3	Comp Het	11	68321729	c.731+4T>C	Splice site mutation					rs766092322<br>0.000009/1 (ExAC)	0.000004<br>(no hom)	-	-	-	-
			11	68367943	c.2173G>C	p.Glu725Gln	Glu	Glu	Glu	Glu	rs2298702<br>0.0013/161 (ExAC)<br>0.0028/14 (1000G)<br>0.00007/2 (TOPMED)	0.001307<br>(4 hom)	DC	Bn<br>(0.015)	Tol<br>(0.06)	Neu<br>(0.323)
	CEP152	Comp Het	15	49033797	c.4093+1G>T	Splice site mutation					rs137967275<br>0.00009/11 (ExAC)<br>0.0002/1 (1000G)	0.000119<br>(no hom)	-	-	-	-
			15	49048668	c.2777A>T	p.Glu926Val	Glu	Glu	Glu	Glu	rs117557829<br>0.0006/75 (ExAC)<br>0.0010/5 (1000G)	0.000548<br>(1 hom)	PM	Dam<br>(0.915)	Del<br>(0.01)	Del<br>(0.811)
	MYH8	Comp Het	17	10302192	c.3874C>T	p.Arg1292*	Arg	Arg	Arg	Arg	rs150008607<br>0.0006/72 (ExAC)<br>0.0014/7 (1000G)<br>0.00007/2 (TOPMED)	0.000666<br>(1 hom)	DC	-	-	-
			17	10310061	c.2117G>A	p.Arg706His	Arg	Arg	Arg	Arg	rs150351713<br>0.0005.65 (ExAC)<br>0.0012/6 (1000G)<br>0.00003/1 (TOPMED)	0.000631<br>(1 hom)	DC	Dam<br>(0.936)	Del<br>(0.01)	Del<br>(0.787)
	ASB12	Hemi	X	63445298	c.233G>A	p.Arg78His					rs145118752<br>0.0002/13 (ExAC)<br>0.0011/4 (1000G)<br>0.00009/1 (GO-ESP)<br>0.00003/1 (TOPMED)	0.000177<br>(no hom,<br>9 hemi)	DC	Bn<br>(0.071)	Tol<br>(0.13)	Neu<br>(0.258)
	RLIM	Hemi	X	73812633	c.518C>G	p.Gln173Glu	Gln	Gln	Gln	Gln	rs186217955<br>0.0003/27 (ExAC)<br>0.0011/4 (1000G)	0.000280<br>(no hom,<br>22 hemi)	DC	Bn<br>(0.084)	Tol<br>(0.11)	Neu<br>(0.278)
	GPR101	Hemi	X	136112845	c.989A>C	p.Asn330Thr	Asn	Ser	Ser	Asn	No	0.000017<br>(no hom,<br>1 hemi)	PM	Bn<br>(0.015)	Tol<br>(0.19)	Neu<br>(0.050)
NIH17A8239849	*NEB*	Comp Het	2	152408302	c.19894A>G	p.Lys6632Glu	Lys	Lys	Lys	Lys	rs765189771<br>0.00004/5 (ExAC)	0.000020<br>(no hom)	PM	Dam<br>(0.627)	Tol<br>(0.19)	Neu<br>(0.268)
			2	152427081	c.17048C>G	p.Ala5683Gly	Ala	Met	Met	Met	No	No	PM	Dam<br>(0.795)	Tol<br>(0.47)	Neu<br>(0.309)
			2	152550904	c.1829G>A	p.Gly610Glu	Gly	Gly	Gly	Gly	rs139125293<br>0.00003/4 (ExAC)<br>0.0002/1 (1000G)	0.000043<br>(no hom)	DC	Dam<br>(1)	Del<br>(0.02)	Del<br>(0.883)
	BAIAP3	Comp Het	16	1388653	c.208G>A	p.Asp70Asn	Asp		Asp	Asp	rs775509338<br>0.00004/2 (ExAC)	0.000032<br>(no hom)	PM	Bn<br>(0.001)	Tol<br>(0.1)	Neu<br>(0.286)

			16	1394080	c.1549C>T	p.Arg517Cys	Arg	Arg	Gly	His	rs762484237<br>0.00002/2 (ExAC)	0.000012<br>(no hom)	DC	Dam<br>(0.528)	Tol<br>(0.06)	Del<br>(0.500)
NIH17A8738324	*ATP8B2*	Hom	1	154321382	c.3460C>T	p.Arg1154Cys	Arg	Arg	Arg	Arg	rs41308375<br>0.0039/441 (ExAC)<br>0.0034/17 (1000G)<br>0.0005/7 (GO-ESP)<br>0.0002/7 (TOPMED)	0.003771<br>(9 hom)	DC	Dam<br>(0.987)	Del<br>(0)	Del<br>(0.881)
	UGT2A3	Comp Het	4	69796345	c.1223G>A	p.Gly408Glu	Gly		Gly		rs139309909<br>0.0006/71 (ExAC)<br>0.0030/15 (1000G)<br>0.00003/1 (TOPMED)	0.000633<br>(1 hom)	DC	Dam<br>(1)	Del<br>(0.02)	Del<br>(0.883)
			4	63796934	c.1023delA	p.Lys341fs	Lys		Lys		rs147333514<br>0.0002/2 (GO-ESP)<br>0.0002/6 (TOPMED)	0.000032<br>(no hom)	-	-	-	-
	HPGDS	Comp Het	4	95220712	c.519G>C	p.Arg173Ser	Arg	Lys	Arg	Arg	rs140417639<br>0.0003/42 (ExAC)<br>0.0012/6 (1000G)<br>0.00003/1 (TOPMED)	0.000307<br>(no hom)	DC	Bn<br>(0.181)	Tol<br>(0.18)	Neu<br>(0.073)
			4	95229787	c.334A>G	p.Lys112Glu	Lys	Lys	Lys	Lys	rs150456822<br>0.0003/42 (ExAC)<br>0.0012/6 (1000G)<br>0.00003/1 (TOPMED)	0.000307<br>(no hom)	DC	Dam<br>(0.994)	Del<br>(0)	Del<br>(0.897)
	ZFAT	Comp Het	8	135614310	c.1652C>A	p.Ala551Asp	Ala	Val	Ala	Ala	rs199682848<br>0.0001/15 (ExAC)<br>0.0006/3 (1000G)	0.000096<br>(no hom)	-	-	-	-
			8	135649769	c.383C>T	p.Thr128Met	Thr	Thr	Pro	Thr	rs143917098<br>0.0001/15 (ExAC)<br>0.0010/5 (1000G)<br>0.00008/1 (GO-ESP)<br>0.00003/1 (TOPMED)	0.000090<br>(no hom)	-	-	-	-
	TNKS1BP1	Comp Het	11	57068427	c.5060C>T	p.Ser1687Leu	Ser	Ser	Ser	Ser	rs554711702<br>0.0002/29 (ExAC)<br>0.0004/2 (1000G)	0.000209<br>(no hom)	DC	Dam<br>(0.998)	Del<br>(0)	Del<br>(0.919)
			11	57077181-<br>57077183	c.3002_3004delAGA	p.Lys1001del					rs747117006<br>0.0002/29 (ExAC)	0.000242<br>(no hom)	-	-	-	-
	TMTC1	Comp Het	12	29786124	c.1084G>A	p.Val362Ile	Val	Val	Val	Val	rs150572401<br>0.0010/122 (ExAC)<br>0.0024/12 (1000G)<br>0.00003/1 (TOPMED)	0.001026<br>(2 hom)	PM	Bn<br>(0.018)	Tol<br>(0.26)	Neu<br>(0.035)
			12	29936611	c.74C>A	p.Ala25Glu		Val	Ala	Ala			PM	Bn<br>(0.148)	Tol<br>(0.18)	Neu<br>(0.069)
	KRT35	Comp Het	17	39635970	c.535G>A	p.Ala179Thr	Ala	Ala	Ala	Ala	rs771101998<br>0.00003/4 (ExAC)	0.000032<br>(no hom)	DC	Dam<br>(0.633)	Del<br>(0.05)	Del<br>(0.567)
			17	39636961	c.389G>A	p.Arg130His	Arg	Arg	Arg	Arg	rs185963879<br>0.0016/192 (ExAC)<br>0.0008/4 (1000G)<br>0.0003/4 (GO-ESP)<br>0.0003/9 (TOPMED)	0.001897<br>(2 hom)	PM	Bn<br>(0.219)	Del<br>(0.02)	Neu<br>(0.405)
	SIGLEC11	Comp Het	19	50461730	c.1461G>C	p.Trp487Cys	Trp		Trp		rs751230929<br>0.000009/1 (ExAC)	0.000004<br>(no hom)	DC	Dam<br>(1)	Del<br>(0.01)	Del<br>(0.905)
			19	50461924	c.1339G>T	p.Val447Phe	Val		Val		rs749024800<br>0.00006/7 (ExAC)<br>0.00003/1 (TOPMED)	0.000041<br>(no hom)	PM	Bn<br>(0.057)	Del<br>(0.02)	Neu<br>(0.387)
	CEP250	Comp Het	20	34091694	c.5497G>A	p.Glu1833Lys	Glu	Glu	Glu	Glu	rs201724904<br>0.0003/31 (ExAC)<br>0.0002/1 (1000G)	0.000228<br>(no hom)	-	-	-	-
			20	34096811	c.6946C>T	p.Arg2316Cys	Arg	Arg	Arg	Arg	No	No	-	-	-	-
	LAMA5	Comp Het	20	60892479	c.7433C>T	p.Ala2478Val	Ala	Ala			rs144781761<br>0.0006/63 (ExAC)<br>0.0012/6 (1000G)	0.000468<br>(1 hom)	PM	Bn<br>(0.082)	Del<br>(0.05)	Neu<br>(0.351)

											0.0003/4 (GO-ESP)<br>0.0001/3 (TOPMED)					
			20	60911479	c.2240G>A	p.Arg747Gln	Arg	Arg	Arg	Arg	rs201992387<br>0.00008/9 (ExAC)<br>0.0006/3 (1000G)	0.000069<br>(no hom)	DC	Dam<br>(0.991)	Tol<br>(0.06)	Del<br>(0.766)
NIH1705180563	*FAT2*	Comp Het	5	150924212	c.6476T>C	p.Val2159Ala	Val	Val	Val	Val	rs201382441<br>0.0002/26 (ExAC)<br>0.0008/4 (1000G)<br>0.00003/1 (TOPMED)	0.000242<br>(no hom)	DC	Dam<br>(0.846)	Del<br>(0)	Del<br>(0.770)
			5	150945824	c.2669T>C	p.Ile890Thr	Ile	Ile	Ile	Ile	rs145215828<br>0.0003/31 (ExAC)<br>0.0008/4 (1000G)	0.000211<br>(1 hom)	PM	Bn<br>(0.186)	Tol<br>(0.41)	Neu<br>(0.033)
	SORBS3	Comp Het	8	22422866	c.902A>G	p.Lys301Arg		Lys		Lys	rs548045720<br>0.0002/4 (ExAC)<br>0.0006/3 (1000G)	0.000136<br>(1 hom)	DC	Bn<br>(0.04)	Tol<br>(0.15)	Neu<br>(0.243)
			8	22426781	c.1426C>T	p.Arg476Cys	Arg	Arg	Arg	Arg	rs540462491<br>0.0004/42 (ExAC)<br>0.0006/3 (1000G)	0.000254<br>(no hom)	DC	Dam<br>(0.592)	Del<br>(0)	Del<br>(0.649)
	KRTAP5-8	Hom	11	71249558	c.457T>C	p.Cys153Ser					No	No	PM	Bn<br>(0.001)	Del<br>(0.01)	Neu<br>(0.406)
	CD163L1	Comp Het	12	7521564-<br>7521567	c.4040-6_4040-<br>3delTCTC	Splice site mutation					No	No	-	-	-	-
			12	7551129	c.1460G>A	p.Gly487Glu	Gly	Gly	Gly	Gly	rs141153568<br>0.00002/2 (ExAC)	0.000026<br>(no hom)	DC	Dam<br>(1)	Del<br>(0)	Del<br>(0.945)
	OVCH1	Comp Het	12	29598250	c.2842G>A	p.Gly948Ser	Gly		Gly		rs745853862<br>0.00002/3 (ExAC)	0.000033<br>(no hom)	-	-	-	-
			12	29614918	c.2149C>T	p.Gln717*	Gln		Gln	Gln	No	0.000008<br>(no hom)	-	-	-	-

YID182829	*PIK3CB*	Hom	3	138433432	c.1180T>G	p.Leu394Val	Leu	Leu	Leu	Leu	rs769366263<br>0.00002/2 (ExAC)	0.000014<br>(no hom)	DC	Dam<br>(0.874)	Tol<br>(0.08)	Del<br>(0.639)
	ASIC3	Hom	7	150748004	c.973G>A	p.Ala325Thr	Ala	Ala	Ala	Ala	rs571908824<br>0.00003/4 (ExAC)<br>0.0002/1 (1000G)	0.000026<br>(1 hom)	PM	Bn<br>(0.014)	Tol<br>(0.05)	Neu<br>(0.346)
	TAF1L	Comp Het	9	32631907	c.3671T>C	p.Phe1224Ser	Phe				rs761527217<br>0.00007/9 (ExAC)<br>0.00005/6 (TOPMED)	0.000087<br>(no hom)	DC	Dam<br>(0.54)	Del<br>(0)	Del<br>(0.627)
			9	32635336	c.242C>T	p.Thr81Met	Thr				rs764175922<br>0.00007/9 (ExAC)<br>0.00006/7 (TOPMED)	0.000090<br>(no hom)	DC	Dam<br>(0.985)	Tol<br>(0.09)	Del<br>(0.728)
	ADAM8	Hom	10	135084732	c.1346C>T	p.Ala449Val	Ala	Ala	Ala	Ala	rs147448426<br>0.0005/50 (ExAC)<br>0.0008/4 (1000G)<br>0.0009/12 (GO-ESP)<br>0.0004/49 (TOPMED)	0.000403<br>(no hom)	DC	Dam<br>(0.993)	Del<br>(0)	Del<br>(0.895)
	NFE2L1	Comp Het	17	46136735	c.2051G>A	p.Arg684His	Arg	Arg	Arg	Arg	rs140314190<br>0.0002/21 (ExAC)<br>0.0005/6 (GO-ESP)<br>0.0002/30 (TOPMED)	0.000148<br>(no hom)	DC	Dam<br>(0.899)	Tol<br>(0.14)	Del<br>(0.605)
			17	46136777	c.2093G>A	p.Arg698Gln	Arg	Arg	Arg	Arg	rs182192213<br>0.00006/7 (ExAC)<br>0.0004/2 (1000G)<br>0.00003/4 (TOPMED)	0.000033<br>(no hom)	DC	Dam<br>(0.758)	Del<br>(0.01)	Del<br>(0.686)
	RBM12	Hom	20	34242574-<br>34242582	c.663_671delTCCTG<br>TGCC	p.Val223_Pro225del					rs541533000<br>0.0004/2 (1000G)<br>0.00002/2 (TOPMED)	0.000477<br>(no hom)	-	-	-	-
	COL18A1	Comp Het	21	46912627	c.2754C>T	Splice site mutation					rs199910738<br>0.0002/18 (ExAC)<br>0.0002/1 (1000G)<br>0.00007/9 (TOPMED)	0.000158<br>(no hom)	-	-	-	-
			21	46927475	c.3757C>T	Splice site mutation					rs756868239<br>0.000009/1 (ExAC)	0.000012<br>(no hom)	-	-	-	-
	PPEF1	Hemi	X	18845519	c.1876A>G	p.Ile626Val	Ile	Ile	Ile	Ile	rs2239435<br>0.0002/21 (ExAC)<br>0.0003/1 (1000G)<br>0.0002/19 (TOPMED)	0.000180<br>(no hom)	DC	Dam<br>(0.994)	Tol<br>(0.08)	Del<br>(0.752)
	ASB12	Hemi	X	63445298	c.233G>A	p.Arg78His					rs145118752<br>0.0002/13 (ExAC)<br>0.0011/4 (1000G)<br>0.00009/1 (GO-ESP)<br>0.0001/13 (TOPMED)	0.000177<br>(no hom)	DC	Bn<br>(0.071)	Tol<br>(0.13)	Neu<br>(0.258)
YID632847	*PRDM16*	Comp Het	1	3102863	c.212C>T	p.Pro71Leu							DC	Dam<br>(0.997)	Del<br>(0)	Del<br>(0.911)
			1	3347441	c.3290A>G	p.Asp1097Gly	Glu	Glu	Glu	Glu	rs777907752<br>0.000008/1 (ExAC)<br>0.00002/3 (TOPMED)	0.000018<br>(no hom)	DC	Bn<br>(0)	Tol<br>(0.23)	Neu<br>(0.042)
	SYNE1	Hom	6	152553246	c.20862+5T>C	Splice site mutation					rs753177718<br>0.000008/1 (ExAC)<br>0.00002/3 (TOPMED)	0.000008<br>(no hom)	-	-	-	-
	PWWP2B	Hom	10	134218351	c.347C>T	p.Pro116Leu		Pro		Pro	rs750405972<br>0.0002/14 (ExAC)<br>0.000008/1 (TOPMED)	0.000090<br>(no hom)	DC	Dam<br>(0.9)	Tol<br>(0.08)	Del<br>(0.656)
	ARHGAP11A	Comp Het	15	32929165	c.2191G>T	p.Asp731Tyr	Val	Asn	Asp	Asp	rs757140912<br>0.00002/2 (ExAC)	0.000012<br>(no hom)	PM	Bn<br>(0.054)	Tol<br>(0.07)	Neu<br>(0.323)
			15	32929478	c.2504G>A	p.Arg835His	Arg	Arg	Arg	Arg	rs774973189<br>0.0001/18 (ExAC)<br>0.0001/13 (TOPMED)	0.000188<br>(no hom)	DC	Dam<br>(0.996)	Del<br>(0)	Del<br>(0.906)
	SULT1A2	Hom	16	28604638	c.529C>T	p.Gln177*	Gln	Lys	Gln	Gln	rs779420691<br>0.00002/2 (ExAC)	0.000024<br>(no hom)	DC	-	-	-

Supplementary Table S2. Predicted functional impacts of amino acid changes of ***SLC2A9b***

Mutation	Func. Impact	Assigned Effect	Inversion Barrier	RMSD (Overall)	ΔRMSF
WT			3.0		
M126V	Medium	Structure	>10.0	7.9	7.9
R351W	High	Structure	>10.0	6.4	6.4

Effects of mutations based on molecular dynamics trajectory analysis. RMSD (Overall) is the mean displacement of the residue measured against the initial, refined wild-type model. RMSD (Local) is the change measured against the 12A region around the residue T:Thr, M:Met, V:Val, R: Arg, W:Trp

Supplementary Table S3. Variant filtering process or unsolved cases

**Individual**	**Total sequence reads**	**Matched reads**	**Total number of variants detected**	**Variants with MAF <1% (dbSNP)**	**Variants filtered by 46 control**	**Non-synonymous and splice site variants**	**Located within splice sites**	**Stop codon gained or lost**	**Insertion or Deletion**	**Missense**
NIH17A8004492	64,404,038	63,765,823 (99.0%)	152,116	30,685	18,940	1,412	102	22	113	1,175
NIH17A8239849	68,750,852	68,019,481 (98.9%)	156,924	32,110	19,964	1,455	104	25	126	1,200
NIH17A8568242	64,956,500	64,301,552 (99.0%)	155,946	32,088	19,949	1,422	105	24	129	1,164
NIH17A8738324	69,795,198	69,020,373 (98.9%)	159,037	33,273	20,793	1,438	96	31	111	1,200
NIH1705180563	68,013,252	67,232,710 (98.9%)	157,342	32,234	20,184	1,555	146	17	122	1,270
YID182829	62,413,708	62,114,408 (99.5%)	131,680	20,049	10,892	1,058	102	12	72	872
YID632847	62,429,460	62,141,319 (99.5%)	132,642	20,390	11,066	1,110	136	17	69	888

Supplementary Table S4. Primer information for rs369512758 (*SLC2A9*)

SLC2A9b	p.Met126Val	Forward	CCACAGGACTGTCCCACTTT
		Reverse	TCAGTGACATGCCTCATGGT

Supplementary Figure S1. Sanger confirmation of the novel variant p.M126V of *SLC2A9b*

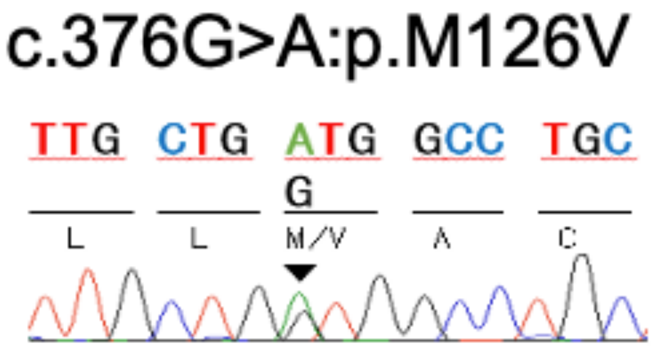

Supplement: Supplementary file 1 [file biomedicines-09-01172-s001.zip › biomedicines-1248799-supplementary.pdf]
